# Overview of the genetic tools in the Archaea

**DOI:** 10.3389/fmicb.2012.00337

**Published:** 2012-10-02

**Authors:** Haruyuki Atomi, Tadayuki Imanaka, Toshiaki Fukui

**Affiliations:** ^1^Department of Synthetic Chemistry and Biological Chemistry, Graduate School of Engineering, Kyoto University, Katsura, Nishikyo-kuKyoto, Japan; ^2^JST, CREST, Sanbancho, Chiyoda-kuTokyo, Japan; ^3^Department of Biotechnology, College of Life Sciences, Ritsumeikan University, Noji-Higashi, KusatsuShiga, Japan; ^4^Department of Bioengineering, Graduate School of Bioscience and Biotechnology, Tokyo Institute of Technology, Nagatsuta, Midori-kuYokohama, Japan

**Keywords:** Archaea, gene disruption, shuttle vectors, genetics, halophiles, methanogens, *Sulfolobus*, Thermococcales

## Abstract

This section provides an overview of the genetic systems developed in the Archaea. Genetic manipulation is possible in many members of the halophiles, methanogens, *Sulfolobus*, and Thermococcales. We describe the selection/counterselection principles utilized in each of these groups, which consist of antibiotics and their resistance markers, and auxotrophic host strains and complementary markers. The latter strategy utilizes techniques similar to those developed in yeast. However, Archaea are resistant to many of the antibiotics routinely used for selection in the Bacteria, and a number of strategies specific to the Archaea have been developed. In addition, examples utilizing the genetic systems developed for each group will be briefly described.

## Introduction

Genetic manipulation, designated here as the ability to introduce, remove, or modify genes in a given organism, is a vital tool to study gene function. Deleting or overexpressing a gene may lead to phenotypic changes that provide valuable clues in determining the physiological role of the gene. Random mutagenesis and the isolation of mutant strains, followed by screening for genes that complement the mutations is a classical strategy to identify groups of genes that are involved in a particular biological function. Genetic manipulation can also be used to engineer cells to improve or introduce a desired function in a cell. The tools necessary for genetic manipulation have been developed in a wide variety of eukaryotes and bacteria, including the yeast *Saccharomyces cerevisiae*, the Gram-negative bacterium *Escherichia coli* and Gram-positive bacterium *Bacillus subtilis*, all of which have been subject to genome-wide gene disruption projects (Giaever et al., 2002; Kobayashi et al., 2003; Baba et al., 2006).

Compared to eukaryotes and bacteria, the development of genetic systems in Archaea is still at a modest stage. Many archaeal species have been found to be resistant against conventional antibiotics utilized for selection in bacterial genetic systems. In addition, many archaeal species can be regarded as extremophiles, preferring growth conditions that greatly differ to those of the mesophilic, aerobic model microbes such as *S. cerevisiae*, *E. coli*, and *B. subtilis*, which adds some difficulty to establish efficient screening methods. For example, when developing a system for hyperthermophilic archaea, the (thermo)stability of the compounds used for selection must also be taken into account, and establishing techniques necessary for growing colonies at high temperatures (and in many cases under an anaerobic environment) are necessary. These factors and others have hampered the development of archaeal genetic systems in the past, but the number of archaea with genetic systems is now increasing at a steady rate. Among the Crenarchaeotes, genetic manipulation is possible in a number of species in the genus *Sulfolobus*. In the Euryarchaeota, genetic systems have been developed in a number of halophiles, methanogens, and members of the Thermococcales. This section will give an overview of the genetic systems developed in these archaeal species, focusing on the principles applied for transformant selection (summarized in Table 1) and some examples of gene disruption that have led to a better understanding of gene function. An in-depth description of the individual organisms and detailed methodology, along with a historical account on the development of these systems, are available in the literature (Whitman et al., 1997; Tumbula and Whitman, 1999; Allers and Mevarech, 2005; Rother and Metcalf, 2005; Berkner and Lipps, 2008; Wagner et al., 2009; Buan et al., 2011; Leigh et al., 2011).

**Table 1:** **A simple summary of the selection strategies employed for genetic manipulation in the Archaea**.

	**Marker gene**	**Host requirements**	**Medium requirements**	**Demonstrated in**			
				**H**	**M**	**S**	**T**
**SELECTION CRITERION**							
Novobiocin resistance	*gyrB* mutant	–	–	O	–	–	–
Mevinolin/simvastatin resistance	*hmgR* overexpression	–	–	O	–	O	O
Puromycin resistance	*pac*	–	–	–	O	–	–
Neomycin resistance	APH3′I/II	–	–	–	O	–	–
Hygromycin B resistance	Thermostable *hph* mutant	–	–	–	–	O	–
Butanol/benzyl alcohol resistance	*adh*	–	–	–	–	O	O
Uracil prototrophy	*pyrE*, *pyrF*	*pyrE^−^*, *pyrF^−^*, *upp*^+^	Pyrimidine-free	O	–	O	O
Leucine prototrophy	*leuB*	*leuB^−^*	Leucine-free	O	–	–	–
Tryptophan prototrophy	*trpE*, *trpAB*	*trpE^−^*, *trpAB^−^*	Tryptophan-free	O	–	–	O
Histidine prototrophy	*hisA*	*hisA^−^*	Histidine-free	–	O	–	–
Lactose prototrophy	*lacS*	*lacS^−^*	Lactose as major carbon/energy source	–	–	O	–
Agmatine prototrophy	*pdaD*	*pdaD^−^*	Applicable with tryptone/yeast extract	–	–	–	O
**COUNTERSELECTION CRITERION**							
5-Fluoroorotic acid resistance	*pyrE*, *pyrF*	*pyrE^−^*, *pyrF^−^*	–	O	–	O	O
6-Azauracil/8-azahypoxanthine/8-aza-2,6-diaminopurine resistance	*hpt*	Resistant w/o *hpt*	–	–	O	–	–
6-Methylpurine resistance	*hpt*	Resistant w/o *hpt*	–	–	–	–	O

H, halophiles; M, methanogens; S, Sulfolobus; T, Thermococcales. Details are described in the text and referred publications.

## Halophiles

### Systems based on antibiotic resistance

**Figure 1:**
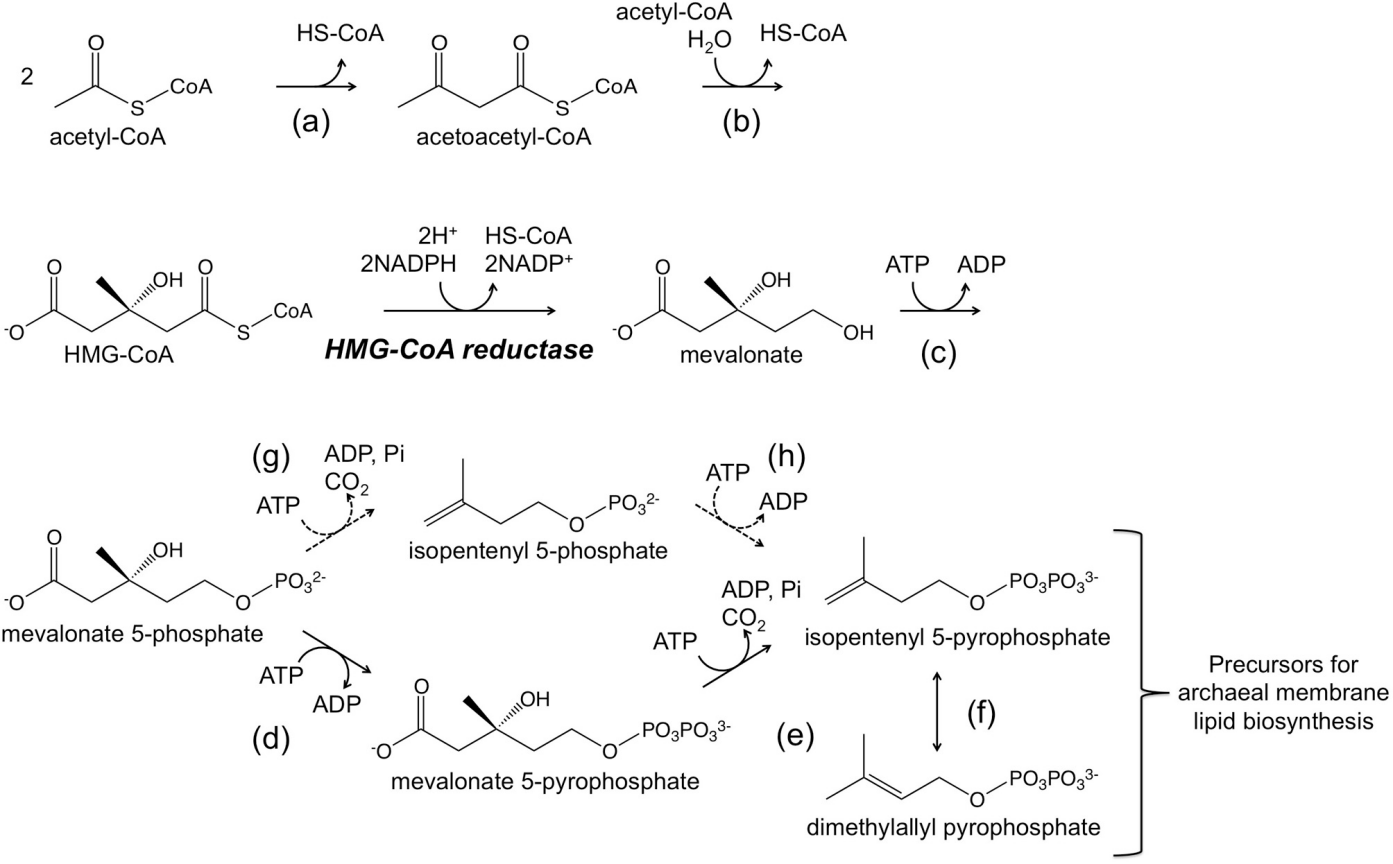
**A schematic illustration of the mevalonate pathway.** The dotted arrows indicate a possible route adopted in the Archaea (Grochowski et al., 2006; Matsumi et al., 2011). Enzymes catalyzing the reactions are (a) acetoacetyl-CoA thiolase, (b) HMG-CoA synthase, HMG-CoA reductase (written), (c) mevalonate kinase, (d) mevalonate-5-phosphate kinase, (e) mevalonate-5-pyrophosphate decarboxylase, (f) isopentenyl-5-pyrophosphate isomerase, (g) mevalonate-5-phosphate decarboxylase, and (h) isopentenyl-5-phosphate kinase.

Genetic systems have mainly been developed in *Halobacterium salinarum* and *Haloferax volcanii*. Systems based on both antibiotic resistance markers and auxotrophic selectable markers have been established. In terms of systems based on antibiotic resistance, novobiocin, which inhibits DNA gyrase, and mevinolin/simvastatin, which inhibits 3-hydroxy-3-methylglutaryl coenzyme A (HMG-CoA) reductase, are two antibiotics that have successfully been applied in halophiles belonging to the genera *Haloferax* and *Halobacterium*. DNA gyrase is a type II topoisomerase that introduces negative supercoils into DNA and whose function is essential for DNA synthesis. HMG-CoA reductase is one of the enzymes of the mevalonate pathway, which utilizes three molecules of acetyl-CoA to synthesize isopentenyl diphosphate (IPP) and its isomer dimethylallyl diphosphate (DMAPP) (Figure 1). IPP and DMAPP are precursors for isoprenoid compounds, which are particularly important for the archaea as their membrane lipids utilize isoprenoid chains. A gene that encodes a novobiocin-resistant DNA gyrase was isolated from *Haloferax* strain Aa2.2 and has been used as a selection marker in developing *Hf. volcanii*–*E. coli* shuttle vectors (Holmes and Dyall-Smith, 1990; Holmes et al., 1991, 1994). Furthermore in *Hf. volcanii*, shotgun cloning of DNA from spontaneous mevinolin-resistant strains led to the isolation of DNA fragments that could transform *Hf. volcanii* to mevinolin resistance, enabling the construction of shuttle vectors (Lam and Doolittle, 1989; Blaseio and Pfeifer, 1990). An examination of various *Hf. volcanii* mevinolin-resistant mutants have revealed that resistance is brought about by either gene amplification or up-promoter mutations, both resulting in enhanced and excess production of HMG-CoA reductase (Lam and Doolittle, 1992). Shuttle vectors such as pWL102 have been shown to also be applicable in transforming members of the genus *Haloarcula* (Cline and Doolittle, 1992). Additional shuttle vectors and gene disruption systems are now available in several members of the *Haloarcula* and their application has been demonstrated (Zhou et al., 2004; Ozawa et al., 2005; Tu et al., 2005).

### Systems based on auxotrophic selectable markers

**Figure 2:**
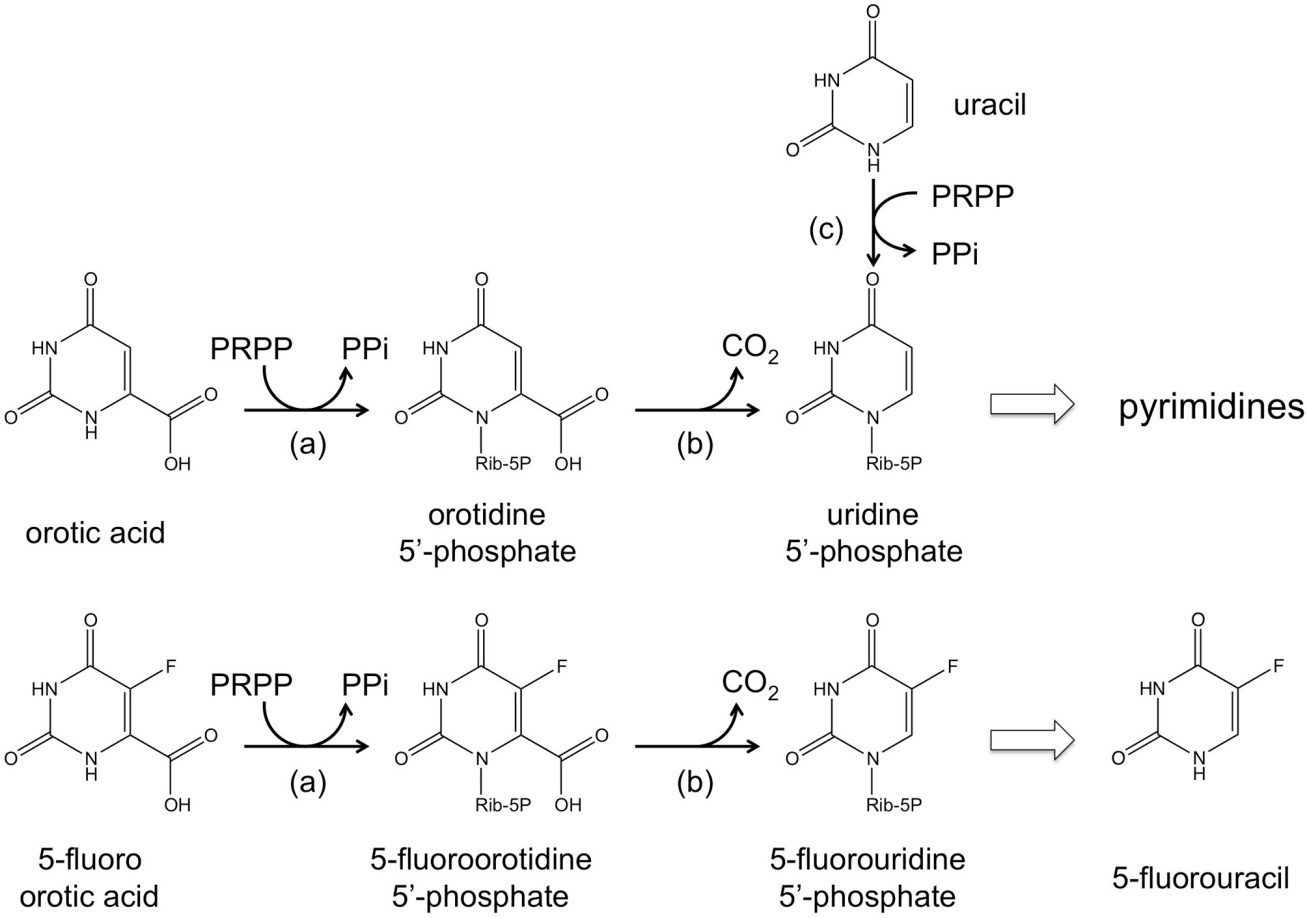
**A schematic illustration of the reactions catalyzed by (a) orotate phosphoribosyltransferase (*pyrE* gene product), (b) orotidine-5′-monophosphate decarboxylase (*pyrF* gene product), and (c) uracil phosphoribosyltransferase (*upp* gene product).** The conversion from 5-fluoroorotic acid (5-FOA) to 5-fluorouridine 5′-phosphate and 5-fluorouracil is also shown.

The *ura3* (or *pyrF*) gene encoding orotidine-5′-monophosphate decarboxylase, an enzyme necessary for *de novo* pyrimidine biosynthesis, has been utilized as a selection marker in a number of halophilic archaea including *Hb. salinarum* NRC-1, *Haloferax mediterranei*, and *Haloarcula hispanica* (Liu et al., 2011). A host cell with a defect in *ura3*/*pyrF* can grow when uracil is added to the medium owing to the function of uracil phosphoribosyltransferase encoded by the *upp* gene. The *ura3*/*pyrF* system is especially convenient as it also allows counterselection. The addition of 5-fluoroorotic acid (5-FOA) to the medium prohibits growth of cells with an intact *ura3*/*pyrF* gene, as 5-FOA is converted to the toxic 5-fluorouridine 5′-phosphate and 5-fluorouracil (Boeke et al., 1984, 1987). These compounds inhibit DNA/RNA synthesis, with the latter known to inhibit thymidylate synthase, an enzyme necessary for thymidine synthesis. It is thus possible to specifically select cells that have lost a *pyrF* (or *pyrE*) gene by supplementing the medium with 5-FOA and a pyrimidine precursor such as uracil (Figure 2). In *Hb. salinarum*, a *ura3*/*pyrF* deletion strain was constructed using a mevinolin resistance marker, and the use of the *ura3*/*pyrF* gene as a counterselection marker has been extensively examined (Peck et al., 2000). Further improvements have enabled the use of *ura3*/*pyrF* as both a selection marker (uracil prototrophy) for initial plasmid integration, and as a counterselection marker (5-FOA resistance) for plasmid excision and gene deletion (Wang et al., 2004). The methodology has been successfully applied in disrupting and examining the arsenic resistance genes of this organism (Wang et al., 2004). The system has also been used to study the physiological roles of TATA binding proteins and transcription factor B proteins, whose genes are present in multiple copies on the genome (Coker and Dassarma, 2007). The methodology developed in *Hb. salinarum* can also be used in *Hf. mediterranei* and *Ha. hispanica*. The system was applied in deleting the phytoene synthase gene in both of these organisms (Liu et al., 2011).

Systems based on other auxotrophic selectable markers have been established in *Hf. volcanii*. The *ura5* (or *pyrE*) gene encoding orotate phosphoribosyltransferase, responsible for the reaction preceding that of the *ura3*/*pyrF* product, has been demonstrated to be applicable as both a selection and counterselection marker (Bitan-Banin et al., 2003). Although two genes (*pyrE1* and *pyrE2*) encoded proteins homologous with PyrE, *pyrE2* was the gene actually involved in pyrimidine biosynthesis. A Δ*pyrE2* strain was constructed and used as a host cell to disrupt the *cmi4* gene of *Hf. volcanii*. Systems based on selection markers involved in amino acid biosynthesis have also been developed in *Hf. volcanii* (Allers et al., 2004). The *leuB* gene, encoding 3-isopropylmalate dehydrogenase in the leucine biosynthesis pathway, and the *trpA* gene that encodes one of the two subunits of tryptophan synthase have been used for selection based on leucine and tryptophan prototrophy, respectively. A convenient system for gene expression has also been developed in *Hf. volcanii* (Allers et al., 2010). Use of the tryptophanase promoter of *Hf. volcanii* (p.*tna*) promoter, which is induced by tryptophan, allows conditional overexpression of the target gene. The genetic background of the host strain has also been modified to facilitate the purification of His-tagged proteins and relieving the need to passage DNA through an *E. coli dam* mutant.

### Application of the genetic systems in halophiles

Gene manipulation is routinely performed in the halophiles and an overwhelming amount of genetic examinations has been reported in the literature (Leigh et al., 2011; Soppa, 2011). This most likely reflects the fact that genetic systems were developed at a relatively early stage in the halophiles and the versatility of the genetic systems themselves, along with the mesophilic and aerobic lifestyles of these organisms. To mention only several of the most recent studies, in *Hb. salinarum*, a gradual inducible gene expression system has been developed (Kixmüller and Greie, 2012). It relies on the promoter of the potassium uptake system operon (P*kdp*), which responds to potassium cation concentrations in the medium. A workflow for genome-wide mapping of transcription factors from *Hb. salinarum* has also been reported (Wilbanks et al., 2012). Target genes such as those encoding the general transcription factor TfbD and the specific transcription factor Bat were modified to incorporate a hemagglutinin tag at the C-termini of the proteins, and the epitopes were used for chromatin immunoprecipitation coupled with high-throughput sequencing. Another study has examined the regulation of bacteriorhodopsin, particularly the relationship between bacterioopsin and retinal biosynthesis (Dummer et al., 2011). The results suggest that bacterioopsin accumulation promotes the production of its cofactor retinal by inhibiting bacterioruberin biosynthesis. In *Hf. mediterranei*, a genetic approach was applied in examining the functions of polyhydroxyalkanoate granule-associated proteins (Cai et al., 2012). The PhaP protein in this organism was found to act as the predominant structure protein on the PHA granules. In *Hf. volcanii*, a conserved archaeal gene with sequence similarity with a tRNA 3′-processing endonuclease has been studied biochemically and genetically, suggesting that in contrary to its annotation, the gene is involved in membrane transport (Fischer et al., 2012). Another study identifies the enzyme responsible for reduction of the ω-position isoprene of dolichol phosphate in *Hf. volcanii* (Naparstek et al., 2012). Single-stranded DNA-binding proteins have also been genetically examined. Five genes that encode proteins homologous to replication protein A (RPA) from *Hf. volcanii* (RpaA1A2, RpaB1B2, RpaC) were analyzed, revealing the essentiality of RpaC and the functional relationship among RPA proteins in this archaeon (Skowyra and Macneill, 2012). A metabolic study identified the enzymes responsible for fructose metabolism. *Hf. volcanii* adopts a bacteria-like phosphoenolpyruvate-dependent phosphotransferase system that generates fructose 1-phosphate, which is further converted to trioses via fructose-1-phosphate kinase and a Class II fructose-1,6-bisphosphate aldolase (Pickl et al., 2012).

## Methanogens

Genetic systems have been developed in a number of species in the genera *Methanococcus* and *Methanosarcina*. DNA-mediated transformation was first demonstrated in *Methanococcus voltae* (Bertani and Baresi, 1987). Most systems in the methanogens rely on antibiotic resistance for selection. Puromycin and the puromycin transacetylase (*pac*) gene from the bacterium *Streptomyces alboniger* (Gernhardt et al., 1990) and its derivatives are often used as the antibiotic and the resistance marker gene, respectively, in methanogen genetics. Initial integration shuttle vectors using the *pac* gene were constructed and successfully used to transform *M. voltae* (Gernhardt et al., 1990; Patel et al., 1994) and *Methanococcus maripaludis* (Sandbeck and Leigh, 1991; Tumbula et al., 1994). Selection based on histidine auxotrophy/prototrophy using the *hisA* gene as a marker has also been demonstrated (Pfeifer et al., 1998). Counterselection methods have been developed (Moore and Leigh, 2005) based on the observation that *M. maripaludis* cells displaying growth were sensitive to the base analogs 6-azauracil and 8-azahypoxanthine (Bowen and Whitman, 1987; Ladapo and Whitman, 1990; Bowen et al., 1996; Kim and Whitman, 1999). Many of the principles and techniques developed in one methanogen have been shown to be applicable in other methanogen species.

In the *Methanosarcina*, efficient introduction of DNA is possible using liposome-mediated transformation (Metcalf et al., 1997). Replicating shuttle vectors were developed for *Methanosarcina acetivorans* and were also found to be applicable in a wide range of other *Methanosarcina* species including *Methanosarcina barkeri*, *Methanosarcina mazei*, and *Methanosarcina thermophila*. Gene disruption using the *pac* gene has been demonstrated in *M. acetivorans* and *M. mazei*. Markerless genetic exchange using the hypoxanthine phosphoribosyltransferase gene (*hpt*) was developed in *M. acetivorans* and also utilized in *M. barkeri*, with counterselection performed based on 8-aza-2,6-diaminopurine (8ADP) resistance (Pritchett et al., 2004; Rother and Metcalf, 2005; Welander and Metcalf, 2008; Buan et al., 2011). An *in vivo* transposon mutagenesis system has also been developed using a modified mariner-family transposable element originally derived from insect (Zhang et al., 2000).

### Application of the genetic systems in methanogens

Using these genetic systems, a number of genes in *M. mazei* Gö1, including those encoding the GlnK_1_ protein and archaeal histone, have been disrupted. *glnK_1_* disruption revealed that GlnK_1_ is not directly involved in the transcriptional regulation of nitrogen assimilation/fixation genes, but does play a role in growth under nitrogen limiting conditions (Ehlers et al., 2005). Disruption of the histone gene was not lethal, but resulted in impaired growth on methanol and trimethylamine, and increased sensitivity to UV light. A broad genome-wide defect in gene transcription was also observed (Weidenbach et al., 2008). In *M. acetivorans*, the *pylT* gene encoding the tRNA for pyrrolysine was disrupted. The disruptant did not show growth defects when grown on methanol or acetate, but could not grow on methylamines, consistent with the fact that the methyltransferases from this organism that are involved in methylamine-dependent methanogenesis possess pyrrolysine (Mahapatra et al., 2006). A genetic approach was also used to distinguish the physiological roles of two gene clusters on the *M. acetivorans* genome encoding an archaeal A_1_A_0_-ATPase and a bacterial F_1_F_0_-ATPase. A mutant disrupted of the latter gene cluster did not display growth defects, and intracellular ATP levels were identical to those in wild-type cells, indicating that the F_1_F_0_-ATPase is dispensible for growth in *M. acetivorans* (Saum et al., 2009). The four studies introduced here have all utilized the *pac* gene for selection of the gene disruptants.

For *M. voltae*, protoplasts can efficiently be transformed by natural or electroporation-mediated uptake of exogenous DNA (Patel et al., 1994). Liposome-mediated transformation has also been applied (Heinicke et al., 2004; Chaban et al., 2009). Gene disruption has been demonstrated on the selenium-free Vhc and Frc hydrogenase genes in order to examine the individual roles of four hydrogenase gene clusters (Berghöfer and Klein, 1995). Four genes encoding the chromatin proteins histone (*hstA*, *hstB*), histone-like protein (*hmvA*), and an Alba homolog (*AlbA*) have been individually disrupted, revealing their involvement in regulation of gene expression (Heinicke et al., 2004). A genetic approach has also been taken to study post-translational protein modification. For example, two genes designated as *aglC* and *aglK* were shown to be necessary for proper *N*-glycosylation in this organism. It was suggested that the two genes are involved in the biosynthesis or transfer of diacetylated glucuronic acid within the glycan structure (Chaban et al., 2009).

In *M. maripaludis*, integration shuttle vectors and methods for auxotroph isolation (see above), and transposon insertion mutagenesis (Blank et al., 1995) and random insertional mutagenesis (Kim and Whitman, 1999) were developed at an early stage (Whitman et al., 1997; Tumbula and Whitman, 1999; Leigh et al., 2011). In addition to puromycin, *M. maripaludis* was found to be sensitive to neomycin, and the use of aminoglycoside phosphotransferase genes APH3′I and APH3′II as selectable markers has been demonstrated (Argyle et al., 1996). Transformation methods have been optimized and are performed via a polyethylene glycol-mediated method (Tumbula et al., 1994). A shuttle vector that replicates in both *E. coli* and *M. maripaludis* was constructed based on the plasmid pURB500 from this archaeon (Tumbula et al., 1997). Using the histone promoter from *M. voltae*, vectors for overexpression of endogenous and heterologous genes have been developed (Gardner and Whitman, 1999). A genetic approach has been taken to examine a wide variety of biological functions in *M. maripaludis*. The mechanisms and regulation of nitrogen fixation has been extensively examined. Repressor binding sites of *nifH*, encoding the nitrogenase reductase component of the nitrogenase complex, have been identified using a *nifH* promoter-*lacZ in vivo* reporter system (Cohen-Kupiec et al., 1997). A similar sequence was found upstream of the glutamine synthetase gene (*glnA*) and shown to function in repression. The repressor protein, NrpR, was identified, and its gene disruption, along with *in vitro* binding experiments, clearly demonstrated its function as a DNA-binding transcriptional repressor that regulates genes involved in nitrogen assimilation (Lie and Leigh, 2003). Further studies have revealed how NrpR binds to specific operator sequences and how it is released from DNA by 2-oxoglutarate binding (Lie et al., 2005, 2010). Furthermore, mechanisms governing posttranslation regulation, namely ammonia switchoff, of nitrogenase have also been examined in detail (Kessler and Leigh, 1999; Kessler et al., 2001; Dodsworth and Leigh, 2006). Genetics have also been utilized to study the energy-conserving hydrogenases in *M. maripaludis*. Gene disruption of one of the two membrane-bound hydrogenase complexes, Ehb, has revealed that the complex is involved in anabolic CO_2_ assimilation (Porat et al., 2006). Results of phenotypic analyses suggested that Ehb donates the electrons necessary for aromatic amino acid biosynthesis from aryl acids via the function of indolepyruvate oxidoreductase (Major et al., 2010). In addition to studies in *M. voltae*, *N*-glycosylation has also been examined in *M. maripaludis*. A putative acetyltransferase gene was subjected to gene disruption, and the mutant cells were found to produce flagellin proteins with sizes corresponding to proteins with defects in glycosylation. In addition to flagellar filament assembly, defects in pilus anchoring were also observed, indicating that flagellum and pilus assembly are linked in their post-translational modification mechanisms (Vandyke et al., 2008). Further studies have identified multiple genes that are necessary for piliation and have also led to the identification of the protein that corresponds to the major pilin monomer, a protein whose gene resides outside of the gene cluster that had been predicted to harbor most of the genes related to pilus formation (Ng et al., 2011). A number of recent studies have examined mechanisms of selenocysteine (Sec) biosynthesis in various strains of *M. maripaludis* (Stock et al., 2010, 2011; Hohn et al., 2011). A selenophosphate synthetase homolog (*selD*) in *M. maripaludis* S2 could not be deleted unless a bacterial selenophosphate synthetase gene was present *in trans*, whereas disruption of the corresponding gene in *M. maripaludis* JJ was possible. Further genetic examination on the latter strain indicated that selenophosphate is the selenium donor in this strain (Stock et al., 2010). In another strain *M. maripaludis* Mm900, which is related to S2, *selD* disruption was possible. Whereas the ability to grow on formate was abolished, hydrogenotrophic growth was unaffected (Hohn et al., 2011). Interestingly, disruption of genes encoding phosphoseryl-tRNA^Sec^ kinase and phosphoseryl-tRNA:Sec-tRNA synthase was possible only when either *selD* was disrupted or if selenium-free hydrogenases were expressed. Detailed biochemical characterization of the gene disruption strains suggests a complex regulatory mechanism of Sec biosynthesis in *M. maripaludis*.

## Sulfolobus

There are many natural genetic elements related to the crenarchaeal genus *Sulfolobus*, including viruses, cryptic plasmids, and transposons (Zillig et al., 1996, 1998; Prangishvili et al., 1998; Stedman et al., 2000). Several transformation systems based on these natural elements were developed in *Sulfolobus solfataricus* and *Sulfolobus acidocaldarius* at an early stage, followed by the establishment of gene manipulation systems based on useful selectable markers (Aagaard et al., 1996; Elferink et al., 1996; Berkner and Lipps, 2008; Wagner et al., 2009; Leigh et al., 2011). Transformation is now mainly carried out by electroporation. In *S. solfataricus*, selection is possible by using a strain with a deletion in *lacS*, which encodes a β-galactosidase. By using an intact *lacS* as a marker gene, transformants can be selected by their ability to grow in a minimal medium containing lactose (Worthington et al., 2003). LacS^+^ colonies can be further identified by blue/white detection using X-Gal (Schelert et al., 2004). Selection based on resistance toward hygromycin B has also been reported using a gene encoding a thermostabilized hygromycin phosphotransferase from *E. coli* (Cannio et al., 1998). Another system is based on resistance toward butanol or benzyl alcohol using an alcohol dehydrogenase gene from *S. solfataricus* (Aravalli and Garrett, 1997). In *S. acidocaldarius* and *Sulfolobus islandicus*, host strains with defects in *pyrE*, *pyrF* or both are utilized, with intact *pyrE* and *pyrF* genes as selection markers (Deng et al., 2009; She et al., 2009; Wagner et al., 2009). This strategy has also been utilized in *S. solfataricus*. Based on these selection strategies, a wide range of *Sulfolobus*–*E. coli* shuttle vectors have been developed and are described in detail in the literature (Aravalli and Garrett, 1997; Stedman et al., 1999; Jonuscheit et al., 2003; Albers et al., 2006; Aucelli et al., 2006; Berkner et al., 2007; Berkner and Lipps, 2008).

### Application of the genetic systems in *Sulfolobus*

#### Sulfolobus solfataricus

Gene disruption based on *lacS* selection has been utilized to examine a wide range of functions in *S. solfataricus*. Conditions for gene disruption have been carefully examined and optimized (Albers and Driessen, 2007). Genetic and biochemical examination has been performed on genes involved in mercury resistance, demonstrating that the *merR* gene product represses transcription of an operon that includes the mercuric reductase gene *merA* (Schelert et al., 2004, 2006). Another study demonstrated that the *copR* gene product is a transcriptional activator of genes encoding copper-transporting ATPase and copper-binding protein and is necessary for copper tolerance of *S. solfataricus* (Villafane et al., 2011). A Lrp-like regulator, Ss-LrpB, has been shown to act as an activator of genes including the pyruvate:ferredoxin oxidoreductase gene (Peeters et al., 2009). A genetic study has also been performed on a heat-shock-inducible ribonucleolytic toxin, VapC6, and its antitoxin VapB6. Analysis of disruption strains of these genes has identified possible targets of the ribonucleolytic activity (Maezato et al., 2011). Genetic manipulation is now also possible for the virus *Sulfolobus* turreted icosahedral virus (STIV) (Snyder et al., 2011; Wirth et al., 2011). An infectious clone of STIV was constructed, and gene disruptions of individual open reading frames and their effects on viral replication have been demonstrated.

#### Sulfolobus acidocaldarius

In *Sulfolobus acidocaldarius*, a series of small multicopy, non-integrative shuttle vectors have been developed and their use in overexpression of genes has been demonstrated (Berkner et al., 2007). Promoters for both constitutive and inducible gene expression have been examined. As for constitutive gene expression, the *sac7d* promoter led to the highest levels of β-galactosidase activity when various promoters were fused upstream of *lacS*. The *mal* promoter was the most suitable for induction, displaying a 17-fold increase upon addition of maltose or dextrin (Berkner et al., 2010). In terms of gene disruption, *pyrE*-deficient host cells have been used with an intact, heterologous *pyrE* from *S. solfataricus* to disrupt putative genes involved in UV photoproduct repair (Sakofsky et al., 2011). It should be noted that in this study, the lengths of the homologous regions flanking the selection marker were approximately 50 bp, introduced by PCR in the primer sequences, which may allow high-throughput gene disruption in a genome-wide scale. Another study clarified two *in vivo* activities of Y-family DNA polymerase in *S. acidocaldarius* (Sakofsky et al., 2012). One activity promotes slipped strand events within simple repetitive sequences and the other promotes insertion of C opposite a potentially miscoding form of G, which may contribute in preventing G:C to T:A transversions. Genetics have contributed in the identification of sulfolobicins, antimicrobial proteins produced by *Sulfolobus* species (Ellen et al., 2011). Antimicrobial tests, protein separation, followed by MS led to the identification of candidate genes, and their disruption confirmed that two genes encoding secretion proteins corresponded to the sulfolobicin. Several studies report a genetic examination of genes involved in cell surface structure of *S. acidocaldarius*. Deletion of individual flagellin genes indicated that all genes were essential for flagellin assembly and that assembly proceeds through hierarchical protein interaction (Lassak et al., 2012). Another study has led to the identification of sulfoquinovose synthase, which is necessary for the synthesis of sulfoquinovose, a component of the *N*-linked glycans on the surface-layer glycoprotein of *S. acidocaldarius* (Meyer et al., 2011). Gene disruption confirmed this activity and also demonstrated the importance of *N*-glycosylation under conditions of increased salt concentrations. A complete genetic analysis of the three type IV pili-like structures in *S. acidocaldarius*, the flagellum, the UV-induced pili, and the adhesive pili, has also been reported (Henche et al., 2012). The effects of single, double, and triple deletion of the three structures on cell surface structure, surface attachment capability, motility, and biofilm formation were examined. It should be noted that this study utilizes cells expressing a codon adjusted, heat stable green fluorescent protein eCGP123. eCGP123 was used to distinguish strains within a biofilm generated from a mixture of strains with different gene deletions. Another study genetically demonstrates that the UV-inducible type IV pili are involved in intercellular, UV-inducible DNA exchange, a valuable mechanism to maintain chromosome integrity (Ajon et al., 2011).

#### Sulfolobus islandicus

In *Sulfolobus islandicus*, several genetic studies have been reported focusing on genes involved in DNA replication and maintenance of DNA topology. In one study, the topoisomerase III gene of *S. islandicus* was disrupted (Li et al., 2011a). Cells were viable but displayed various defects in chromosome distribution, cell size, and gene transcription. The results suggested that this enzyme plays an important role in chromosome segregation and maintenance of DNA topology for gene transcription. Another study addressed whether any of the three proliferating cell nuclear antigen (PCNA) on the *S. islandicus* genome are dispensable or not (Zhang et al., 2010). Disruption strains could not be isolated for any of the genes, and an improved knockout system has been described in order to carefully examine the essentiality of each gene. A recent study reported the development of a new gene disruption system in *S. islandicus* that is based on antibiotic resistance toward simvastatin (Zhang and Whitaker, 2012). The selectable marker gene was a construct promoting overexpression of the HMG-CoA reductase gene.

## Thermococcales

Genetic systems have mainly been developed in *Thermococcus kodakarensis* and *Pyrococcus furiosus*. Gene disruption has also been demonstrated in *Thermococcus onnurineus* (Kim et al., 2010). Shuttle vectors are available for *Pyrococcus abyssi* (Lucas et al., 2002). In *T. kodakarensis*, gene disruption was accomplished by using host strains deleted of the *pyrF* and/or *trpE* genes, and selection with the corresponding intact marker gene (Sato et al., 2003, 2005). A system has also been developed based on the simvastatin/HMG-CoA reductase overexpression system (Matsumi et al., 2007). An improved system utilizing host cells that exhibit agmatine auxotrophy due to deletion of the arginine decarboxylase gene (Fukuda et al., 2008) has also been developed (Santangelo et al., 2010). This system allows selection in complex media and not only accelerates the gene disruption procedure, but should also contribute to the isolation of mutant cells that require nutrient-rich conditions for cell growth. Counterselection is performed with 5-FOA in the *pyrF* system (Sato et al., 2005), and counterselection in nutrient-rich medium is possible using a hypoxanthine–guanine phosphoribosyltransferase gene which, when present, results in 6-methylpurine sensitivity (Santangelo et al., 2010). In *Pyrococcus furiosus*, two transformation systems based on shuttle vectors that replicate in *P. furiosus* and *E. coli* have been developed. One is based on the shuttle vector system pYS2 from *P. abyssi*. The selectable marker is an HMG-CoA reductase overexpression cassette, and selection is based on resistance toward simvastatin (Waege et al., 2010). The other system is based on the *P. furiosus* chromosomal origin and utilizes the *pyrF* gene as a selectable marker in combination with the *P. furiosus* COM strain (Δ*pyrF*). The plasmids existed in a single copy in *P. furiosus*, and were stable without selective pressure for more than 100 generations (Farkas et al., 2011). The *pyrF* deletion strain *P. furiosus* COM1 can also be used as an efficient host for gene disruption (Lipscomb et al., 2011). This strain displays natural competence and a remarkable efficiency in DNA uptake, allowing marker replacement using linear as well as circular DNA. Using *pyrF* as the selectable marker, construction of markerless deletion mutants via counterselection by 5-FOA resistance has been demonstrated (Lipscomb et al., 2011). In a recent report, the limits of recombination efficiency of *P. furiosus* COM1 have been examined. It was found that marker replacement was possible with as few as 40 nucleotides of flanking homology to the target region (Farkas et al., 2012), which will surely facilitate genetic studies in this organism. Markerless deletion was utilized to disrupt the *trpAB* genes, encoding the two subunits of tryptophan synthase. The disruptant displayed tight tryptophan auxotrophy, and the wild-type *trpAB* genes could be used as a selectable marker in this strain (Farkas et al., 2012).

### Application of the genetic systems in thermococcales

#### Thermococcus kodakarensis and thermococcus onnurineus

**Figure 3:**
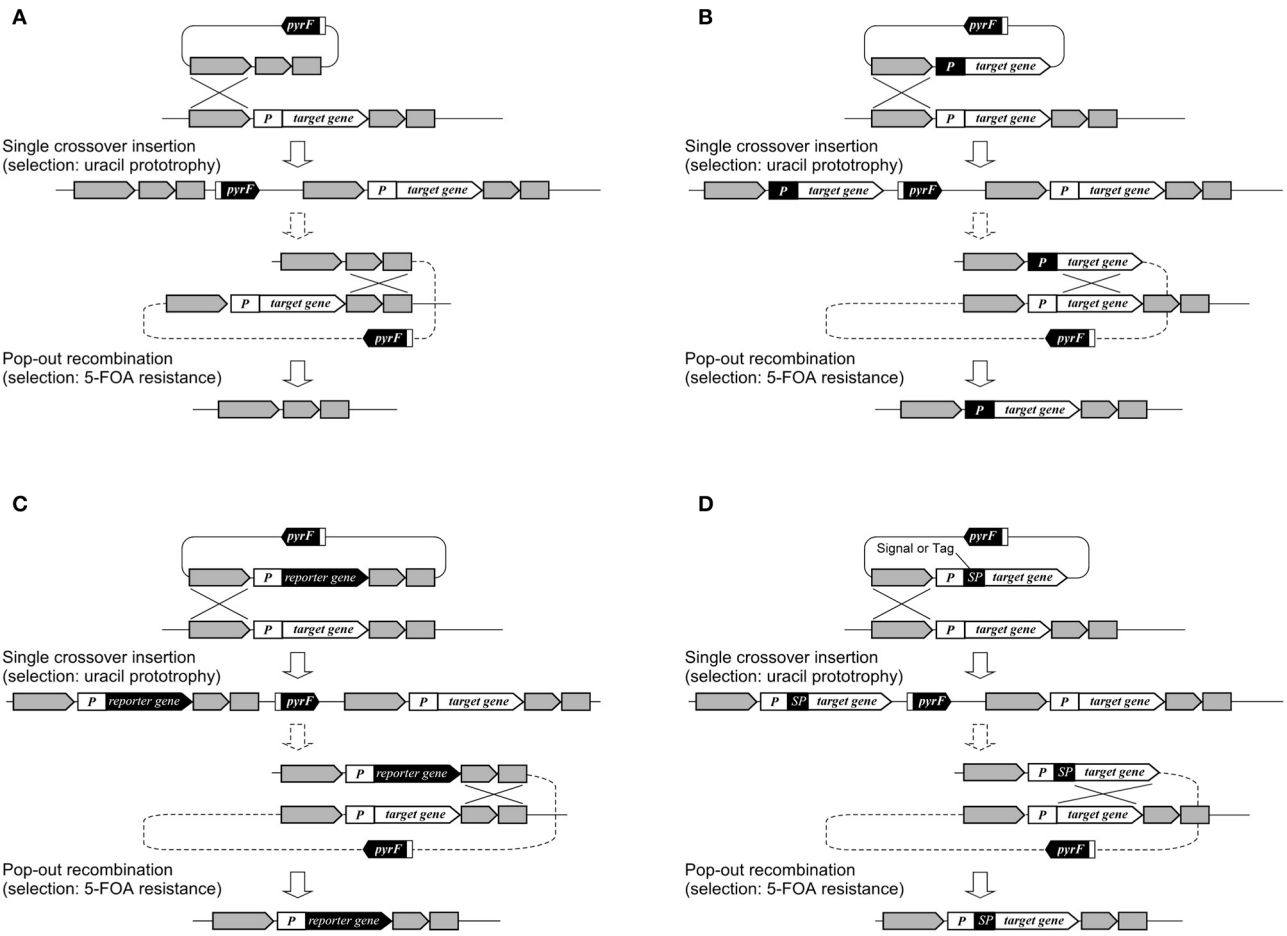
**Various applications using selection/counterselection marker genes. (A)** Use in markerless gene disruption; **(B)** use in promoter exchange; **(C)** use in reporter gene integration; and **(D)** use in signal peptide (SP) or tag integration. The use of *pyrF* is shown, but other selection/counterselection marker genes can be applied in a similar manner in a wide range of Archaea.

In *T. kodakarensis*, a wide range of genes has been disrupted in order to understand their physiological functions such as those involved in transcription and its regulation, DNA replication, and metabolism. The functions of individual transcription factors such as TFB1/2 (Santangelo et al., 2007), RNA polymerase subunits E and F (Hirata et al., 2008), the switch 3 loop of subunit B (Santangelo and Reeve, 2010) have been examined, along with sequences that can promote transcription termination (Santangelo et al., 2009). Deletion of transcription regulator genes, followed by transcriptome analysis, has led to the identification of regulons and the function of these regulators (Kanai et al., 2007, 2010). In terms of DNA replication, a large number of proteins expected to be involved in DNA replication were His-tagged. Protein complexes were isolated and their components identified, revealing the various protein networks involved in DNA replication (Li et al., 2010). This led to the discovery of a novel GINS-associated nuclease, GAN (Li et al., 2011b). In other studies, genetic analyses of three *mcm* genes on the genome have revealed the essentiality/dispensability of the individual genes (Ishino et al., 2011; Pan et al., 2011). Disruption of the reverse gyrase gene indicated that the enzyme provides an advantage to cells when grown at temperatures of 80°C or higher (Atomi et al., 2004). In terms of metabolism, genes examined include those involved in glycolysis (Imanaka et al., 2006; Matsubara et al., 2011), gluconeogenesis (Sato et al., 2004), pentose metabolism (Orita et al., 2006), as well as coenzyme A (Yokooji et al., 2009), polyamine (Morimoto et al., 2010), and compatible solute biosynthesis (Borges et al., 2010). Gene disruption of three putative hydrogenase gene clusters and phenotypic analyses indicated that the cytosolic hydrogenase Hyh and the membrane-bound oxidoreductase complexes Mbh and Mbx are involved in H_2_ consumption, H_2_ generation, and H_2_S generation, respectively (Kanai et al., 2011). Disruption of various pathways related to hydrogen production/consumption has clarified the reductant flux in *T. kodakarensis* and has demonstrated strategies to elevate hydrogen production in this organism (Santangelo et al., 2011). *T. kodakarensis* harbors two pairs of genes encoding chaperonins and prefoldins, and gene disruption has been performed to distinguish the functions of the individual proteins at different temperatures (Danno et al., 2008; Fujiwara et al., 2008; Gao et al., 2012). One of multiple homologs of NAD(P)H oxidase has been disrupted to examine its relationship with the oxygen sensitivity of this anaerobic archaeon (Kobori et al., 2012). Systems for gene overexpression, tagging, and protein secretion have been established (Santangelo et al., 2008; Mueller et al., 2009; Yokooji et al., 2009; Takemasa et al., 2011), in many cases relying on the selection/counterselection strategy utilizing the *pyrF* marker gene (Figure 3). In *T. onnurineus*, gene disruption has been demonstrated using the simvastatin/HMG-CoA reductase overexpression system. Gene disruption led to the identification of the gene cluster encoding formate hydrogen lyase, cation/proton antiporter and formate transporter, which were responsible for the growth of this organism on formate (Kim et al., 2010).

#### Pyrococcus furiosus

Using the shuttle vector pYS3, the RNA polymerase subunit D gene with a HisTag sequence was expressed with an inducible promoter deriving from the fructose-1,6-bisphosphatase gene from *P. furiosus*. This allowed a simple two-step purification of the thermostable RNA polymerase from this organism (Waege et al., 2010). In the process of developing the shuttle vector system based on the *P. furiosus* chromosomal origin, the minimum replication origin sequence required for autonomous plasmid replication in this organism has been identified (Farkas et al., 2011). Interestingly, the *cdc6/orc1* gene adjacent to *oriC* was not required in *cis* for replication of the shuttle vector in *P. furiosus*. The gene disruption system developed with *P. furiosus* COM1 has been successfully applied in disrupting individual or double gene disruptions of two cytoplasmic hydrogenase genes (Lipscomb et al., 2011). The system was further applied for detailed genetic studies on proteins related to elemental sulfur metabolism, membrane-bound oxidoreductase complex (Mbx), cytoplasmic coenzyme A-dependent NADPH:sulfur oxidoreductase (Nsr), and sulfur-induced protein A (SipA) (Bridger et al., 2011). The *mbx* disruptant displayed growth defects in the presence of sulfur, and little, if any, sulfide generation was observed, demonstrating that Mbx plays a critical role in elemental sulfur reduction and energy conservation in *P. furiosus*. Gene manipulation has also been used to overexpress the cytoplasmic [NiFe]-hydrogenase SHI. The promoter of the PF1399 gene, which encodes the S-layer protein, was fused upstream of the four-gene operon (PF0891–PF0894) encoding SHI. In the overexpression strain, a 20-fold higher SHI transcript level was observed, and moreover, a 100-fold higher amount of hydrogenase was obtained when compared with the highest homologous [NiFe]-hydrogenase system previously reported (Chandrayan et al., 2012). In another study, the lactate dehydrogenase gene from the moderately thermophilic *Caldicellulosiruptor bescii* was introduced into *P. furiosus* under the control of the cold-induced protein A (*cipA*, PF0190) promoter. Transcript levels of *cipA* in *P. furiosus* are 26-fold higher in cells grown at 72°C compared to those grown at 98°C. This recombinant strain, when grown at 98°C, ferments sugar to produce acetate and hydrogen as end products, as is the case of wild-type *P. furiosus*. When grown at 72°C, however, the strain generates lactate at concentrations up to 3 mM, demonstrating a temperature-dependent regulation of metabolism (Basen et al., 2012).

## Future perspectives

The genetic systems developed in the halophiles, methanogens, *Sulfolobus*, and Thermococcales provide the tools to carry out sophisticated genetic analyses in these organisms (Leigh et al., 2011). With the abundance of genome sequence information, functional genomics in these organisms is a realistic approach. On the other hand, the Archaea comprise a diverse group of organisms, and there are still many interesting organisms that cannot be examined genetically. Genetics are limited to *Sulfolobus* in the Crenarchaeota, and considering the wealth of genome sequence information, the development of genetic systems in *Pyrobaculum* and *Thermoproteus* would greatly promote research in these genera. Genetic tools for *Ignicoccus* can be considered crucial to understand its unique relationship with *Nanoarchaeum equitans*. In the Euryarchaeota, the thermophilic/acidophilic Thermoplasmatales and the sulfate-reducing Archaeoglobales are major orders in which genetic tools are still not available. Another major group is the (hyper)thermophilic methanogens. Although much needs to be done, the variety of tools that have been developed will surely provide a basis to explore the possibilities of developing genetic systems in other archaea.

### Conflict of interest statement

The authors declare that the research was conducted in the absence of any commercial or financial relationships that could be construed as a potential conflict of interest.
